# Geant4 Simulation of the Effect of Different Composites on Polyimide Photon and Neutron Shielding Properties

**DOI:** 10.3390/polym15081973

**Published:** 2023-04-21

**Authors:** Hanan Akhdar, Rawan Alotaibi

**Affiliations:** Department of Physics, Faculty of Science, Imam Mohammad Ibn Saud Islamic University (IMSIU), Riyadh 11623, Saudi Arabia

**Keywords:** Polyimide, Geant4, composites, shielding properties, photon, neutron

## Abstract

Polymers are widely used materials that have many medical and industrial applications. Some polymers have even been introduced as radiation-shielding materials; therefore, many studies are focusing on new polymers and their interactions with photons and neutrons. Research has recently focused on the theoretical estimation of the shielding effectiveness of Polyimide doped with different composites. It is well known that theoretical studies on the shielding properties of different materials through modeling and simulation have many benefits, as they help scientists to choose the right shielding material for a specific application, and they are also much more cost-effective and take much less time compared to experimental studies. In this study, Polyimide (C_35_H_28_N_2_O_7_) was investigated. It is a high-performance polymer, well known for its outstanding chemical and thermal stability, as well as for its high mechanical resistance. Because of its exceptional properties, it is used in high-end applications. The performance of Polyimide and Polyimide doped with different weight fractions of composites (5, 10, 15, 20 and 25 wt.%) as a shielding material against photons and neutrons were investigated using a Monte Carlo-based simulation toolkit Geant4 within a wide range of energies of both photons and neutrons from 10 to 2000 KeVs. Polyimide can be considered a good neutron shielding material, and its photon shielding abilities could be further enhanced when adding different high atomic number composites to it. The results showed that Au and Ag gave the best results in terms of the photon shielding properties, while ZnO and TiO_2_ had the least negative effect on the neutron shielding properties. The results also indicate that Geant4 is a very reliable tool when it comes to evaluating the shielding properties against photons and neutrons of any material.

## 1. Introduction

The characteristics of polymers have made them excellent choices for a wide range of applications since they are inexpensive, flexible, and simple to handle. Recently, scientists have examined the photon and neutron-shielding characteristics of various polymers to assess how well they can attenuate radiation, particularly those with specific applications in radiation-related utilities. Much of this research demonstrated that polymers’ photon attenuation capabilities are promising, and their capacity to attenuate photons be boosted by combining them with various composites with high atomic numbers. Numerous investigated polymers also have high thermal neutron cross-sections and high neutron particle sensitivity, making them excellent neutron-shielding materials [1,2,3,4,5].

Polyimides (C_35_H_28_N_2_O_7_) are a class of high-performance polymers well known for their outstanding chemical and thermal stabilities, as well as for their high mechanical properties [6].

Recent studies have indicated that polymers integrated with composites have a high potential to provide reasonable protection against radiation. In this study, the effect of selected composites on Polyimide is investigated theoretically.

Aluminum, iron oxide, aluminum, zinc oxide, and titanium dioxide composites were chosen as the most significant composites that have outstanding impacts on polymers’ shielding properties. Silver and gold composites also have a good impact on polymers’ properties, but it is well known that silver and gold composites are expensive; that is why experimental studies of their effects are limited, and a better approach is to use simulation to examine the properties of a substance doped with silver or gold composites.

In this work, photon and neutron attenuation properties of Polyimide doped with different weights fractions of those mentioned composites (5, 10, 15, 20, 25 wt.%) at a large energy range (from 10 to 2000) keV have been theoretically investigated using Monte Carlo simulation.

## 2. Materials and Methods

### 2.1. Materials

**Figure 1:**
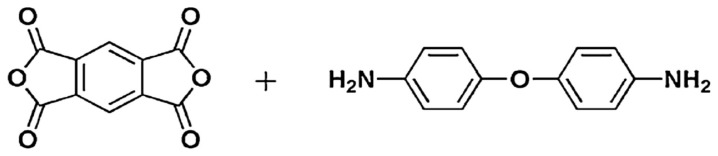
Polyimide repeating unit. (Adapted with permission from [8]. Copyright 2012, Elsevier).

Polyimides (C_35_H_28_N_2_O_7_) are a class of high-performance polymers well known for their outstanding chemical and thermal stabilities, as well as for their high mechanical properties [6]. Figure 1 shows the repeating unit of Polyimide, which has a density of 1.2 g/cm^3^. Because of Polyimides’ superior thermal, mechanical, and dielectric qualities, Polyimides have been widely used in the microelectronics industry as materials for electronic packaging and electrical insulation. Functional Polyimides and their uses have recently gained attention as new study areas, particularly in the areas of polymer electronic memories, flexible displays, flexible solar devices, and high-performance interlayer dielectrics [7,8].

### 2.2. Photon Attenuation

The photon attenuation characteristics of the shielding material can be investigated by determining different variables. These variables include linear attenuation coefficients, mass attenuation coefficients, HVLs, effective atomic numbers and effective electron densities [9].

Any beam of photons experiences attenuation by photoelectric absorption, Compton scattering, and pair production when a material of a certain thickness is being traversed [10]; Equation (1) is used to calculate the photon mass attenuation coefficient (*μ_m_*) [11]:

(1) $$I=I_{0}e^{-\mu_{m}x}$$

where (*I*_0_) is the un-attenuated and (*I*) is the intensity of the attenuated photon after passing through a mass per unit area (*x*) layer of a sample. Equation (2) is used to calculate the linear attenuation coefficient (*μ*):

(2) $$\mu =\mu_{m}\rho$$

where (ρ) is the density of the sample’s material [11,12]. Another important parameter is the half-value layer (HVL) of the material, which could be found using Equation (3) [11,12]:

(3) $$HVL=\frac{\ln2}{\mu }$$

The total molecular cross-section can be found using Equation (4) [13,14]:

(4) $$\sigma_{t,m}=\left(\frac{\mu }{\rho }\right)\frac{A_{t}}{N_{A}}$$

Moreover, the total atomic cross-section can be calculated by Equation (5) [13,14]:

(5) $$\sigma_{t.a}=\sigma_{t.m}\frac{1}{\sum_{i}^{n}\left(n_{i}\right)}$$

where (N*_A_*) is Avogradro’s number, (A*_t_* = Σ*n_i_*A*_i_*) is the molecular weight, (*A_i_*) is the atomic weight of an element of the compound, and (*n_i_*) is the total number of atoms. While the total electronic cross-section for the element is given by Equation (6) [14]:

(6) $$\sigma_{t.el}=\frac{1}{N_{A}}\sum_{i}^{n}\frac{f_{i}A_{i}}{Z_{i}}{\left(\mu_{mt}\right)}_{i}$$

where (*f_i_*) is the number of atoms of element (*i*) relative to the total number of atoms of all elements in the compound, and (*Zi*) is the atomic number of the *i*th element in the compound. The effective atomic number (*Zeff*) of the compound could be found from the ratio between the total atomic cross-section and the total electronic cross-section given by Equation (7) [13,15]:

(7) $$Z_{e\mathrm{ff}}=\frac{\sigma_{t,a}}{\sigma_{t,el}}$$

While the effective electron density is given by Equation (8) [13,15]:

(8) $$N_{eff}=\frac{\mu_{m}}{\sigma_{t,el}}$$

All these parameters are essential when studying the photon shielding properties of any material, and in this work, they were all investigated theoretically on Polyimide.

### 2.3. Neutron Attenuation

The effective removal cross-section (Σ*_R_*) represents the probability of the neutron reactions within the material and is given by Equation (9) [15]:

(9) $$\Sigma_{R}={\sum_{i}\rho_{i}\left(\Sigma_{R}/\rho \right)}_{i}$$

where (*ρi*) is the partial density, (*Σ_R_*/ρ) is the mass removal cross-section, which can be calculated for any compound by Equation (10) [16]:

(10) ΣRρ=0.206A−13Z−0.294

where (*A*) is the atomic weight and (*Z*) is the atomic number.

The HVL, which is the thickness needed to reduce the neutron intensity to half of its original value, is given by Equation (11) [15,16]:

(11) $$HVL={ln2/\Sigma }_{R}$$

The fast neutron removal cross-section of any element can be calculated using the empirical formulas indicated by Equations (12) and (13) [2]:

Σ_*R*_ = 0.190*Z* − 0.743   *if*
*Z* ≤ 8 (12)

Σ_*R*_ = 0.125*Z* − 0.565   *if*
*Z* > 8 (13)

## 3. Results

### 3.1. Photon Shielding Properties

**Figure 2:**
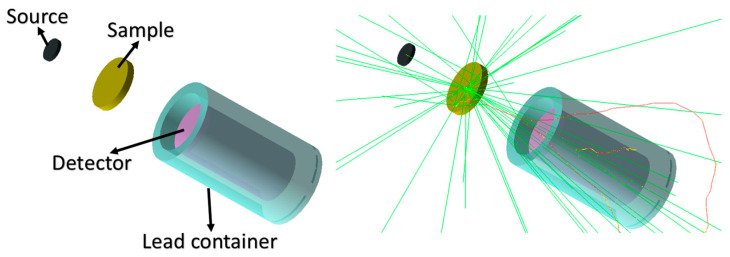
Geant4 simulation code setup.

**Figure 3:**
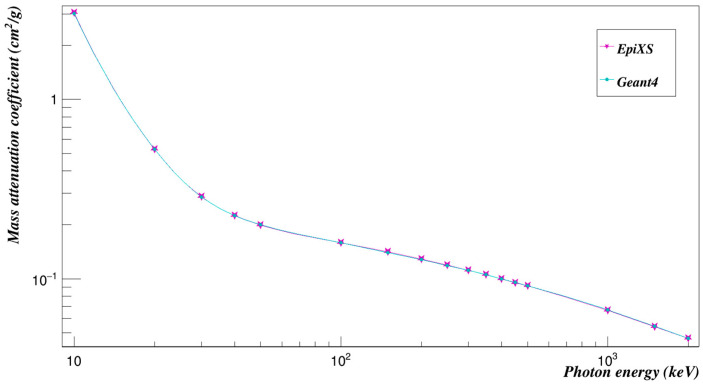
Validation of Geant4 results of the mass attenuation coefficients by those found by EpiXS.

The photon mass attenuation coefficients of the relatively introduced polymer, Polyimide, were investigated at the studied energies using Geant4 [17]. In order to validate the results, they were compared to those from EpiXS [18], which is a Windows-based program for photon attenuation, dosimetry and shielding based on EPICS2017 and EPDL9 database that allows obtaining the photon cross-section data for any sample. Geant4 is a toolkit based on the Monte Carlo statistical method that simulates the passage of particles in matter [17]. Version 10.07 of the Geant4 toolkit, developed by CERN, Switzerland was used in the current work. A simple setup of a sample, a source and a detector were used, as shown in Figure 2. The green lines represent the track of uncharged particles such as photons and neutrons where red lines represent the track of charged particles. The source shoots mono-energetic photons or neutrons in one direction toward the sample. The mass attenuation coefficient was found using the number of photons/neutrons reaching the detector with and without the sample. Figure 3 represents a comparison between the mass attenuation coefficients obtained by Geant4 and EpiXS. The HVLs were also found and compared to those found by EpiXS using Equation (14), as shown in Table 1. The effective atomic numbers and effective electron densities of Polyimide were also found in the studied energy range, as seen in Table 2 and Table 3.

% Δ = 100 × (*µ_EpiXS_* − *µ_G4_*)/*µ_EpiXS_* (14)

### 3.2. Neutron Shielding Properties

The crucial factor in determining a material’s ability to block neutrons is its neutron effective removal cross-section, which is defined as; the likelihood that a fast or fission energy neutron will experience its first collision and thus be eliminated from the group of penetrating neutrons [19]. Table 4 summarizes the neutron effective removal cross-sections of Polyimide as found using Geant4.

### 3.3. Polyimide with Composites

#### 3.3.1. Aluminum

The most often employed composites addition in the creation of polymer composites is aluminum. Their growing popularity is due to the improved thermal and mechanical qualities they offer. For optoelectronic applications, aluminum composites exhibit distinctive optical properties [20,21]. Polyimide was introduced with different weight fractions of aluminum composites (5, 10, 15, 20, and 25 wt.%).

**Figure 4:**
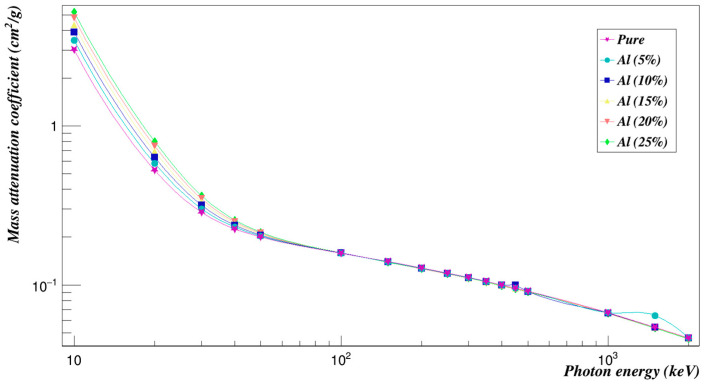
Photon mass attenuation coefficients of Polyimide doped with aluminum.

Figure 4, which was plotted using Root (6.10/04) software, developed by CERN, Switzerland [22], shows the values of the mass attenuation coefficients of aluminum-doped Polyimide for energies from 10 to 2000 keV as found by Geant4.

**Figure 5:**
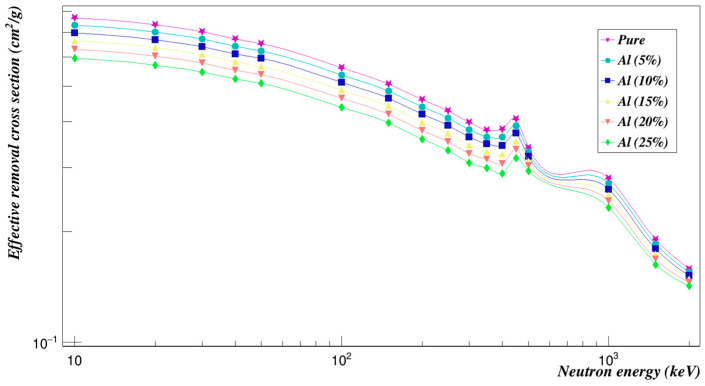
Neutron effective removal cross sections of Polyimide doped with aluminum.

The neutron-effective removal cross-sections were also found and plotted in Figure 5. As can be seen, the neutron effective removal cross-section decreased nonlinearly with the increase of neutron energy for all concentrations. Among the studied concentrations of aluminum composites added to Polyimide, Al composites (25%) loaded to Polyimide showed relatively lower values, and Al composites (5%) loaded with Polyimide had the highest neutron effective removal cross-section.

#### 3.3.2. Iron Oxide

The Polyimide was introduced with different weight fractions of iron oxide composites (5, 10, 15, 20, and 25 wt.%). Fe_3_O_4_ composites have been observed to enhance the magnetic characteristics of polymers. However, the electrical conductivity frequently decreases after the addition of Fe_3_O_4_ composites, but research has focused on it due to its distinct physical or chemical properties and being antibacterial agents [23,24,25].

**Figure 6:**
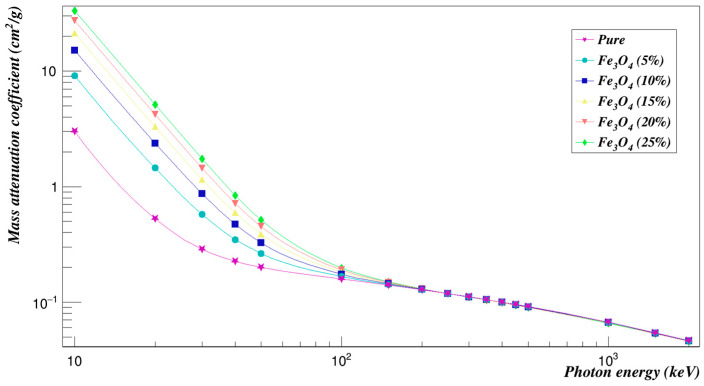
Photon mass attenuation coefficients of Polyimide doped with iron oxide.

The mass attenuation coefficients decrease exponentially with the increase of energy, which agrees with Lambert-Beer law, and this trend means that the attenuation ability of the samples under evaluation is relatively high at low energy, and the samples can shield more photons, while this ability to shield photons reduces as the energy increases. The photoelectric phenomenon clearly affects the attenuation coefficient values at low energy, and this explains the quick decrease observed. Besides, an increase in the attenuation coefficients is observed with an increase in the concentration of composites, as can be seen in Figure 6. This increase occurs since the composites are heavy elements, and it is known that heavy elements are preferable for shielding applications.

**Figure 7:**
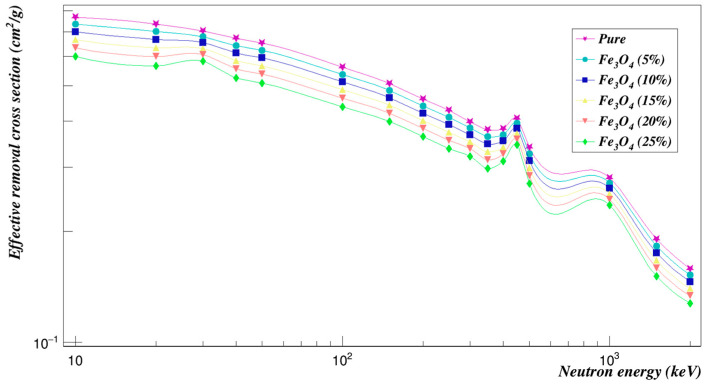
Neutron effective removal cross sections of Polyimide doped with iron oxide.

Figure 7 summarizes the neutron effective removal cross-sections of Polyimide doped with Fe_3_O_4_ found using Geant4. It can be seen that Polyimide doped with iron oxide composites for 5% concentration are the most effective ones among the other concentrations for neutron shielding.

#### 3.3.3. Zinc Oxide

ZnO composites are utilized in polymers to boost surface hardness and corrosion resistance. Enhancing compressive and flexural strengths, as well as reducing shrinkage, have been made possible by ZnO. The most prized properties of ZnO include its physical and chemical stability, photocatalytic activity, absorption of UV and infrared light, antibacterial properties and non-toxicity. The shielding capacity is enhanced by ZnO composites [26,27].

**Figure 8:**
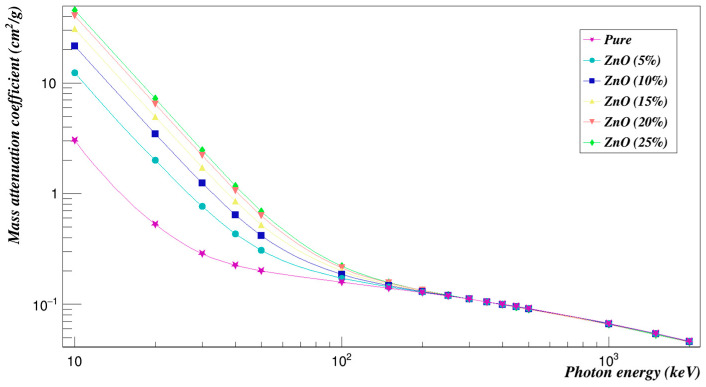
Photon mass attenuation coefficients of Polyimide doped with zinc oxide.

**Figure 9:**
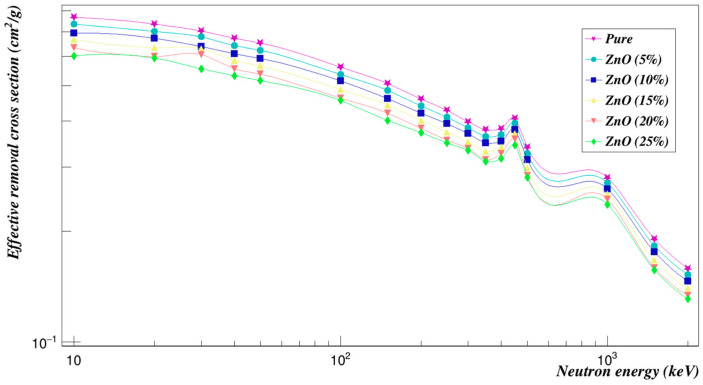
Neutron effective removal cross sections of Polyimide doped with zinc oxide.

Figure 8 shows a plot of the mass attenuation coefficients for each concentration at different energies. It was observed that the mass attenuation coefficient decreases rapidly with increasing the photon energy and increases with increasing the weight fraction of ZnO composites. Figure 9 is a plot of the investigated radiation shielding properties against neutrons. The higher the concentration of zinc oxide composites, the lower the cross-sections of the effective removal of neutrons, as can be seen.

#### 3.3.4. Titanium Dioxide

TiO_2_ composites are used as fillers frequently because they increase chemical stability and have a high level of corrosion resistance without being harmful. They are used to improve the mechanical qualities of polymers. The principal application of titanium dioxide (TiO_2_) is a material modification and the increase of impact resistance [28,29].

**Figure 10:**
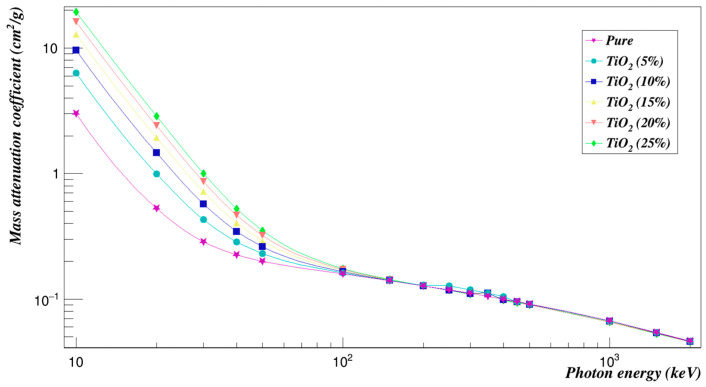
Photon mass attenuation coefficients of Polyimide doped with titanium dioxide.

**Figure 11:**
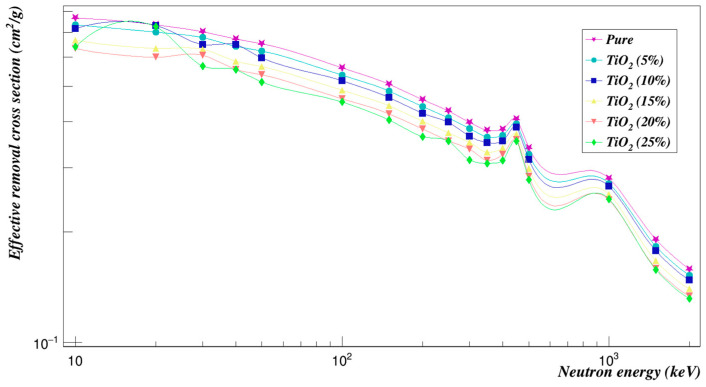
Neutron effective removal cross sections of Polyimide doped with titanium dioxide.

The measured mass attenuation coefficient values of the Polyimide with titanium dioxide for energy from 10 to 2000 keV are plotted in Figure 10. Figure 11 shows the effective removal cross-sections for neutrons, Σ_*R*_ of the Polyimide doped with titanium dioxide composites. It can be seen that the values of the effective removal cross-sections for neutrons at different concentrations of the composites are very close to each other.

#### 3.3.5. Silver

Silver composites are used with polymers to improve their permittivity. Since it is desirable to have a composite with a high dielectric constant, one typically searches for fillers with these properties that are also highly polarizable. The addition of silver composites enhances the material’s physical characteristics, such as flexibility, density, and attenuation coefficient, making it more suitable for photon shielding applications [30,31]. Silver composites are preferred for this reason, alongside additional functional assets such as their ductility, electrical, and thermal conductivity [32].

**Figure 12:**
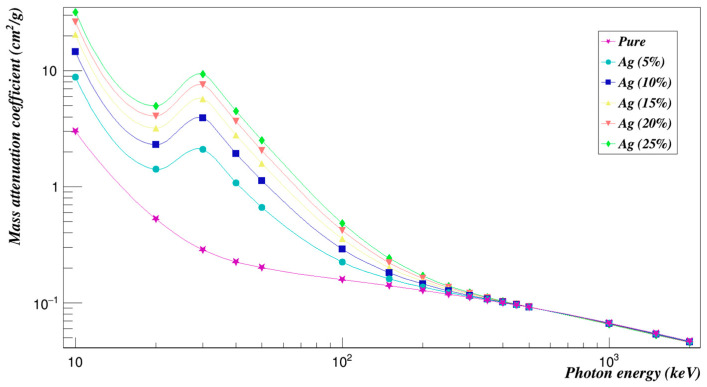
Photon mass attenuation coefficients of Polyimide doped with silver.

Figure 12 shows a trend of the results for the mass attenuation coefficients with respect to photon energy. Mass attenuation coefficients reach high values and diverge in the low energy range. The primary interaction mechanism in this region is the photoelectric process, which is high energy and atomic number sensitive. Up to 1500 keV, the mass attenuation coefficient values dramatically decline and then nearly remain unchanged. This is caused by the pair creation process that predominates above 1500 keV and is dependent on the chemical makeup of the Polyimide doped with silver composites.

**Figure 13:**
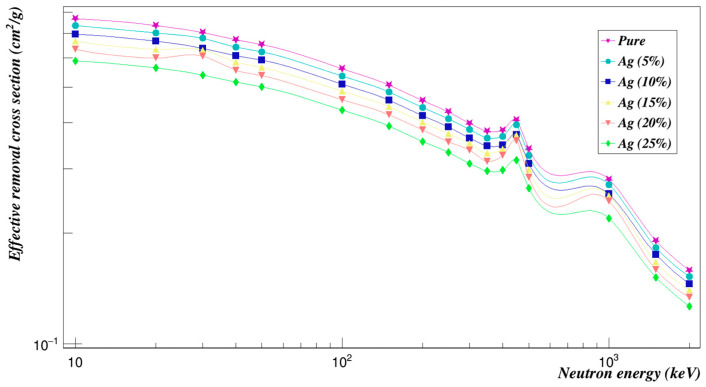
Neutron effective removal cross sections of Polyimide doped with silver.

Figure 13 shows the effective removal cross sections of the Polyimide doped with silver composites. While the concentration of Ag composites increases, the effective neutron removal cross-sections decrease.

#### 3.3.6. Gold

Gold composites are expensive, yet it is simple to manage their size, shape, aspect ratio, and dispersion stability. Polymer-based gold composites have the benefit of being mechanically flexible. When gold composites are added to polymers, flexural strength is increased. Gold composites are also a good choice for fillers because they have desirable properties such as stability, non-toxicity, uniform particle size, and antimicrobial properties [29,33,34].

**Figure 14:**
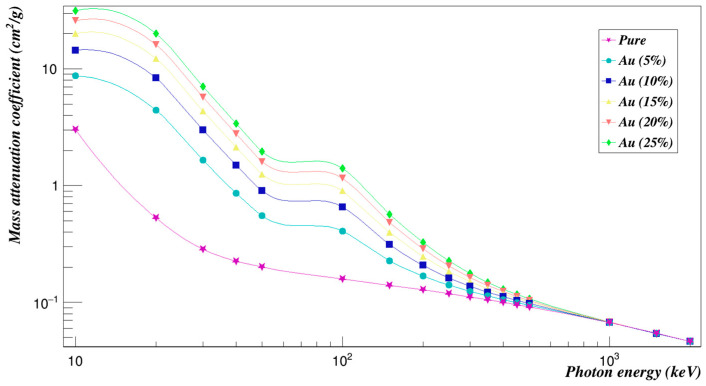
Photon mass attenuation coefficients of Polyimide doped with gold.

**Figure 15:**
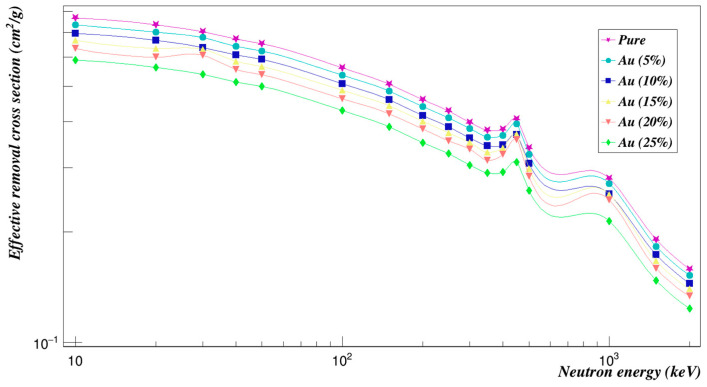
Neutron effective removal cross sections of Polyimide doped with gold.

Figure 14 represents the mass attenuation coefficients with incident photon energy for the Polyimide doped with gold composites in the studied energy range. Obviously, the values of the mass attenuation coefficients for all concentrations decrease with the increase of the photon energy. The Σ_*R*_ values of all concentrations of gold composites added to Polyimide are given in Figure 15. It decreases with increasing concentrations and also with increasing energy.

#### 3.3.7. Comparison between All Studied Composites

**Figure 16:**
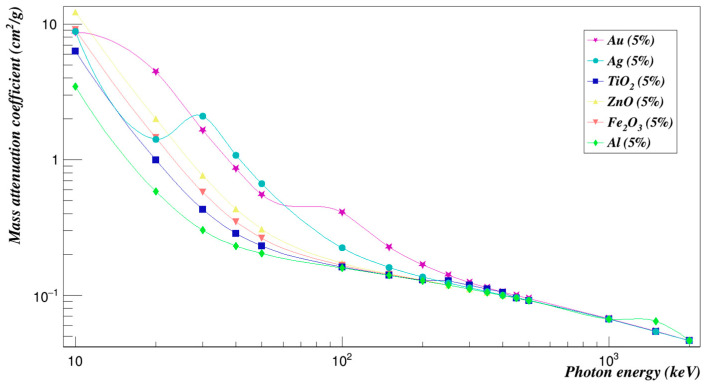
Mass attenuation coefficients of Polyimide doped with 5% of different composites.

**Figure 17:**
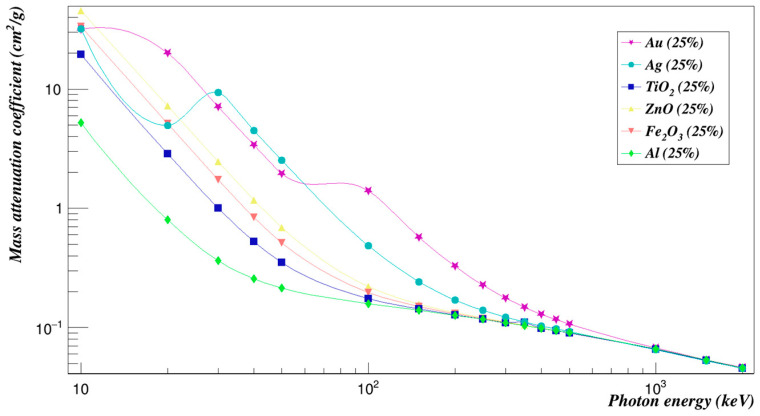
Mass attenuation coefficients of Polyimide doped with 25% of different composites.

**Figure 18:**
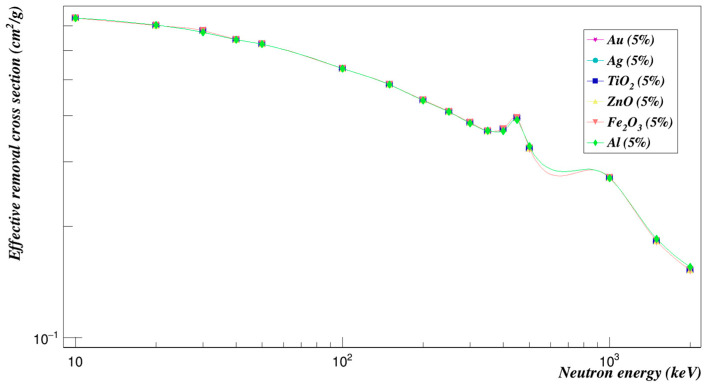
Effective removal cross sections of Polyimide doped with 5% of different composites.

**Figure 19:**
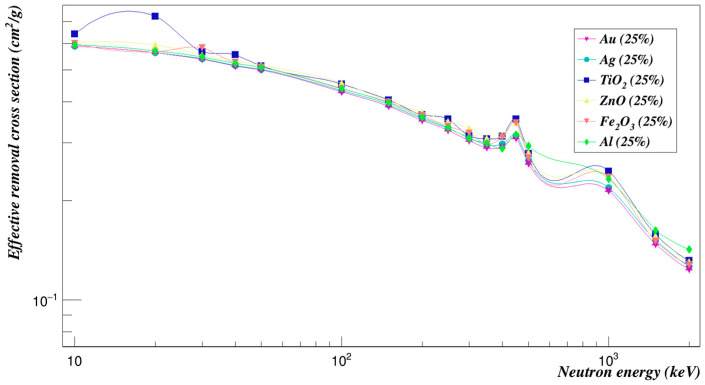
Effective removal cross sections of Polyimide doped with 25% of different composites.

In order to compare the effects of all the different composites that were investigated in this work, the concentration of 5% and 25% of composites added were plotted against each other in Figure 16, Figure 17, Figure 18 and Figure 19. We can see from Figure 16 that at low energies, all studied samples’ mass attenuation coefficients were close except for the Polyimide doped with zinc oxide composites, which increased by 306.32% at a concentration of 5%. Moreover, at the energy of 30 keV, gold and silver composites had increased the mass attenuation coefficient of Polyimide by 633.86% and 474.96%, respectively. At higher energies, results begin to converge for all composites. The effect was studied at a concentration of 25% for all composites added to Polyimide, as shown in Figure 17. Zinc oxide composites have increased the mass attenuation coefficient of Polyimide by 1531.59% at a concentration of 25% at 10 keV energy. Gold composites have increased the mass attenuation coefficient of Polyimide by 836.97% at an energy of 20 keV. Gold and silver composites had the closest and strongest effects at energies between 30 keV and 50 keV. They had increased the mass attenuation coefficient of Polyimide by 3169.28% to 1159.78% for Polyimide doped with gold composites and 2374.80% to 878.54% for Polyimide doped with silver composites. The effect of gold composites had the largest impact on the mass attenuation coefficients at energies between 100 keV and 500 keV, which increased by 206.84% to 0.50%. The Polyimide with aluminum composites added for the mass attenuation coefficient has the lowest mass attenuation coefficient. All composites added to Polyimide converge at high energies.

The same comparison was made on the neutron effective removal cross-sections. Figure 18 shows that at low energies, the Polyimide doped with the titanium dioxide composites has the highest removal cross-section. And at the energy of 30 keV, titanium dioxide composites and iron oxide composites had a greater effect than the rest of the composites. At higher energies, results begin to converge for all composites, and the highest values were for Polyimide doped with titanium dioxide composites, followed by iron oxide, zinc oxide, then aluminum. The lowest values were for Polyimide doped with gold and silver composites.

The comparison between the composites’ effects on the Polyimide was made at a concentration of 25% for all composites, as shown in Figure 19. The neutron effective removal cross-section values were much lower than those with 5% concentrations at low energies. At medium energies, all values converged. At higher energies, the Polyimide doped with titanium dioxide and with zinc oxide had the highest removal cross sections values. The lowest values were for the Polyimide doped with gold and silver composites.

## 4. Discussion

In this work, the photon and neutron shielding properties of Polyimide were studied at the energy range between 10 and 2000 keV. The photon characteristics of Polyimide were investigated utilizing the mass attenuation coefficients, half-value layers, effective atomic numbers, and effective electron densities to show the ability of Polyimide to act as a photon shielding material. Polyimide has excellent shielding performance for neutrons and is also superior to Polyimide doped with various composites. The EpiXS and Geant4 results agree very well.

Using the Geant4 powerful toolkit based on the Monte Carlo method, we examined the photon and neutron shielding capabilities of polymer composite materials based on Polyimide with varied (Al, Fe_3_O_4_, ZnO, TiO_2_, Ag and Au) composite contents with weight fractions of (5, 10, 15, 20, and 25%).

The shielding properties of the Polyimide were very promising, and the addition of different composites enhanced its photon shielding properties. However, it was observed that the effect of the addition of composites on the mass attenuation coefficients of Polyimide is very large at low photon energies, then starts to decrease as the energy increases until it starts to saturate with the coefficients of the pure Polyimide or even slightly decreases. This decrement becomes larger when the weight factor of the composites increases. This means that a compromise must be made when choosing the right amount of composites in order to ensure the increase of attenuation coefficients at certain energy ranges but at the same time considering that the original coefficients do not decrease at high photon energies.

It was observed that Al and TiO_2_ composites enhance the attenuation coefficients to photons up to 100 and 150 keV, respectively, while Fe_3_O_4_ and ZnO up to 250 and 300 KeV, respectively. Ag composites manage to increase the Polyimide attenuation coefficients up to photon energy of 500 keV, while Au composites to photons of 1000 keV before it saturates with the pure Polyimide attenuation coefficients.

Among all studied composites, Al and TiO_2_ were the least to increase the photon mass attenuation coefficients of Polyimide, with an average change in coefficients among all the studied energy range between (2.98%) and (9.47%) for Al and between (18.59%) and (85.6%) for TiO_2_ at the different investigated weight factors (5–25%). Where for Fe_3_O_4_ composites the change was between (33.41%) and (167.03%) and between (53.58%) and (243.63%) for ZnO composites. In contrast, silver and gold composites were the best, with differences between (98.45%) and (492.27%) for silver and between (127.27%) and (636.34%) for gold.

As for the neutron shielding abilities of the Polyimide, it is well known that the addition of high Z composites would decrease its effective removal of cross sections. This decrement increases with the weight factor of the added composites.

Within the studied composites, TiO_2_ and ZnO had the least effect on the removal cross sections as the average percentage difference between the removal cross sections of Polyimide with and without the composites were between (−3.39%) and (−16.96%) and between (−4.09%) and (18.58%) for TiO_2_ and ZnO respectively. Fe_3_O_4_ and Al composites decreased the removal cross sections of neutrons to a range between (−4.04%) and (−20.20%) and between (−4.05%) and (−20.25%), respectively, at different weight factors. Ag and Au had the largest impact on removal cross sections as they were decreased by (−4.47%) to (−22.38%) and (−4.68%) to (−23.39%), respectively.

This study provides a comparison of the effects of different composites on the shielding properties of both photons and neutron Polyimide at a wide range of energies so that researchers who need to work with Polyimide can choose among the composites based on the desired application and how much cost they are willing to provide.

## 5. Conclusions

The shielding properties of the Polyimide were very promising; those properties were enhanced when adding different composites. Adding some composites at high concentrations to Polyimide may not give very good results. Further studies of different composites at energy ranges may be necessary to cover the shielding capabilities of Polyimide fully; indeed, mixing two or more composites with Polyimide may give even better results and have different effects on its shielding properties. The results also indicate that Geant4 is a very reliable tool when it comes to evaluating the shielding properties against photons and neutrons of any material.

## Figures and Tables

**Table 1:** Half value layers of the investigated Polyimide as found by EpiXS and Geant4.

Photon Energy (keV)	HVL (cm)		
	EpiXS	Geant4	% ∆
10	0.19040	0.1918	−0.74%
20	1.09314	1.0942	−0.10%
30	2.01375	2.0197	−0.30%
40	2.56578	2.5672	−0.06%
50	2.89111	2.8809	0.35%
100	3.63350	3.6386	−0.14%
150	4.09191	4.1185	−0.65%
200	4.48423	4.5156	−0.70%
250	4.84129	4.8642	−0.47%
300	5.17144	5.1843	−0.25%
350	5.48591	5.4838	0.04%
400	5.77899	5.7714	0.13%
450	6.06421	6.0431	0.35%
500	6.33206	6.3013	0.49%
1000	8.67631	8.6105	0.76%
1500	10.6646	10.6311	0.31%
2000	12.4339	12.4220	0.10%

**Table 2:** The effective atomic number of the investigated Polyimide was found by EpiXS and Geant4.

Photon Energy (keV)	Effective Atomic Number (Z_eff_)		
	EpiXS	Geant4	% ∆
10	5.81050	6.41677	−10.43%
20	5.57293	5.54240	0.55%
30	5.15411	4.87071	5.50%
40	4.80646	4.59039	4.50%
50	4.61173	4.47689	2.92%
100	4.36176	4.30716	1.25%
150	4.32577	4.26577	1.39%
200	4.31508	4.25744	1.34%
250	4.31026	4.26223	1.11%
300	4.30772	4.27016	0.87%
350	4.30606	4.28162	0.57%
400	4.30493	4.28786	0.40%
450	4.30427	4.29682	0.17%
500	4.30383	4.30007	0.09%
1000	4.30232	4.30941	−0.16%
1500	4.30380	3.39023	21.23%
2000	4.31066	3.38299	21.52%

**Table 3:** The effective electron density of the investigated Polyimide was found by EpiXS and Geant4.

Photon Energy (keV)	The Effective Electron Density N_eff_ (×10^23^ Electrons g^−1^)		
	EpiXS	Geant4	% ∆
10	4.28027	4.72666	−10.43%
20	4.10527	4.08259	0.55%
30	3.79675	3.58781	5.50%
40	3.54065	3.38133	4.50%
50	3.39721	3.29772	2.93%
100	3.21306	3.1727	1.26%
150	3.18656	3.14221	1.39%
200	3.17868	3.13607	1.34%
250	3.17513	3.1396	1.12%
300	3.17325	3.14544	0.88%
350	3.17204	3.15388	0.57%
400	3.17120	3.15848	0.40%
450	3.17071	3.16508	0.18%
500	3.17039	3.16748	0.09%
1000	3.16928	3.17436	−0.16%
1500	3.17037	2.49728	21.23%
2000	3.17542	2.49194	21.52%

**Table 4:** The neutron effective removal cross-sections of Polyimide in the investigated energy range as found using Geant4.

Neutron Energy (keV)	The Neutron Effective Removal Cross-Sections (cm^2^/g)	Neutron Energy (keV)	The Neutron Effective Removal Cross-Sections (cm^2^/g)
10	0.76887	300	0.39868
20	0.73592	350	0.37957
30	0.70413	400	0.38153
40	0.67230	450	0.40726
50	0.65358	500	0.33969
100	0.56134	1000	0.28019
150	0.50720	1500	0.19076
200	0.45903	2000	0.15843
250	0.42839		

## Data Availability

The data presented in this study are available on request from the corresponding author.

## References

[1] Akhdar H. (2022). Theoretical Investigation of Gamma- and Neutron-Shielding Properties of Polysulfone (PSU) Polymer Material Using geant4. Polymers.

[2] Akhdar H., Marashdeh M.W., AlAqeel M. (2022). Investigation of Gamma Radiation Shielding Properties of Polyethylene Glycol in the Energy Range from 8.67 to 23.19 Kev. Nucl. Eng. Technol..

[3] Singh T., Kaur U., Singh P.S. (2010). Photon Energy Absorption Parameters for Some Polymers. Ann. Nucl. Energy.

[4] Mehrara R., Malekie S., Kotahi S.M.S., Kashian S. (2021). Introducing a Novel Low Energy Gamma Ray Shield Utilizing Polycarbonate Bismuth Oxide Composite. Sci. Rep..

[5] Akhdar H. (2022). Theoretical Investigation of Fast Neutron and Gamma Radiation Properties of Polycarbonate-Bismuth Oxide Composites Using geant4. Nanomaterials.

[6] Verny L., Ylla N., Da Cruz-Boisson F., Espuche E., Mercier R., Sudre G., Bounor-Legaré V. (2020). Solvent-Free Reactive Extrusion as an Innovative and Efficient Process for the Synthesis of Polyimides. Ind. Eng. Chem. Res..

[7] Liu Y.-W., Tang L.-S., Qu L.-J., Liu S.-W., Chi Z.-G., Zhang Y., Xu G.-R. (2019). Synthesis and Properties of High Performance Functional Polyimides Containing Rigid Nonplanar Conjugated Fluorene Moieties. Chin. J. Polym. Sci..

[8] Liaw D.-J., Wang K.-L., Huang Y.C., Lee K.R., Lai J.Y., Ha C.S. (2012). Advanced Polyimide Materials: Syntheses, Physical Properties and Applications. Prog. Polym. Sci..

[9] Kaçal M.R., Akman F., Sayyed M.I. (2019). Evaluation of Gamma-Ray and Neutron Attenuation Properties of Some Polymers. Nucl. Eng. Technol..

[10] Mann H.S., Brar G.S., Mudahar G.S. (2016). Gamma-Ray Shielding Effectiveness of Novel Light-Weight Clay-Flyash Bricks. Radiat. Phys. Chem..

[11] Hubbell J.H. (1982). Photon Mass Attenuation and Energy-Absorption Coefficients. Int. J. Appl. Radiat. Isot..

[12] Kaewkhao J., Laopaiboon J., Chewpraditkul W. (2008). Determination of Effective Atomic Numbers and Effective Electron Densities for Cu/Zn Alloy. J. Quant. Spectrosc. Radiat. Transf..

[13] Un A., Faruk D. (2013). Determination of Mass Attenuation Coefficients, Effective Atomic Numbers and Effective Electron Numbers for Heavy-Weight and Normal-Weight Concretes. Appl. Radiat. Isot..

[14] Singh K., Singh H., Sharma V., Nathuram R., Khanna A., Kumar R., Bhatti S.S., Sahota H.S. (2002). Gamma-Ray Attenuation Coefficients in Bismuth Borate Glasses. Nucl. Instrum. Methods Phys. Res. Sect. B Beam Interact. Mater. At..

[15] Olukotun S.F., Mann K.S., Gbenu S.T., Ibitoye F.I., Oladejo O.F., Joshi A., Tekin H.O., Sayyed M.I., Fasasi M.K., Balogun F.A. (2019). Neutron-Shielding Behaviour Investigations of Some Clay-Materials. Nucl. Eng. Technol..

[16] Singh Mann K. (2015). Toolkit for Fast Neutron Removal Cross-Section. Proceedings of the 3rd International Conference Advancements in Engineering and Technology.

[17] Ivanchenko V. (2003). Geant4 toolkit for simulation of HEP experiments. Nuclear Instruments and Methods in Physics Research Section 257 A: Accelerators, Spectrometers. Detect. Assoc. Equip..

[18] Hila F.C., Asuncion-Astronomo A., Dingle C.A.M., Jecong J.F.M., Javier-Hila A.M.V., Gili M.B.Z., Balderas C.V., Lopez G.E.P., Guillermo N.R.D., Amorsolo A.V. (2021). EpiXS: A Windows-based program for photon attenuation, 254 dosimetry and shielding based on EPICS2017 (ENDF/B-VIII) and EPDL97 (ENDF/B-VI.8). Radiat. Phys. Chem..

[19] Uddin Z., Yasin T., Shafiq M., Raza A., Zahur A. (2020). On the Physical, Chemical, and Neutron Shielding Properties of Polyethylene/Boron Carbide Composites. Radiat. Phys. Chem..

[20] Huang X.Y., Jiang P.K., Kim C.U. (2007). Electrical Properties of Polyethylene/Aluminum Nanocomposites. J. Appl. Phys..

[21] Huang X., Kim C., Jiang P., Yin Y., Li Z. (2009). Influence of Aluminum Nanoparticle Surface Treatment on the Electrical Properties of Polyethylene Composites. J. Appl. Phys..

[22] Brun R., Rademakers F. (1997). ROOT—An object oriented data analysis framework. Nucl. Instrum. Methods Phys. Res. Sect. A Accel. Spectrometers Detect. Assoc. Equip..

[23] Wang L., Qiu H., Liang C., Song P., Han Y., Han Y., Gu J., Kong J., Pan D., Guo Z. (2019). Electromagnetic Interference Shielding MWCNT-Fe_3_O_4_@Ag/Epoxy Nanocomposites with Satisfactory Thermal Conductivity and High Thermal Stability. Carbon.

[24] Badawy S.M., Abd El-Latif A.A. (2015). Synthesis and Characterizations of Magnetite Nanocomposite Films for Radiation Shielding. Polym. Compos..

[25] Kubiak A., Kubacka M., Gabala E., Anna D., Karol S., Katarzyna S.C., Katarzyna C., Teofil J. (2020). Hydrothermally Assisted Fabrication of Tio_2_-Fe_3_O_4_ Composite Materials and Their Antibacterial Activity. Materials.

[26] Khalaf M.A., Cheah C.B., Ramli M., Ahmed N.M., Al-Shwaiter A. (2021). Effect of Nano Zinc Oxide and Silica on Mechanical, Fluid Transport and Radiation Attenuation Properties of Steel Furnace Slag Heavyweight Concrete. Constr. Build. Mater..

[27] Abbas M.I., Alahmadi A.H., Elsafi M., Alqahtani S.A., Yasmin S., Sayyed M.I., Gouda M.M., Ahmed M.E. (2022). Effect of Kaolin Clay and Zno-Nanoparticles on the Radiation Shielding Properties of Epoxy Resin Composites. Polymers.

[28] Nikbin I.M., Mohebbi R., Dezhampanah S., Mehdipour S., Mohammadi R., Nejat T. (2019). Gamma Ray Shielding Properties of Heavy-Weight Concrete Containing Nano-tio2. Radiat. Phys. Chem..

[29] Rachel R., Vidhya J., Azhagarasan N.S., Jayakrishnakumar S., Hariharan R. (2022). Evaluation of the Effect of Titanium Dioxide and Gold Nanoparticles Surface Treatment on the Flexural Strength of Polymethyl Methacrylate Heat Cure Denture Base Resin. J. Clin. Adv. Dent..

[30] Gonon P., Boudefel A. (2006). Electrical Properties of Epoxy/Silver Nanocomposites. J. Appl. Phys..

[31] Abdali K. (2021). Crystal Structural, Morphological and Gamma Ray Shielding (γ-S) Efficiency of PVA/PAAM/PAA Polymer Blend Loaded with Silver Nanoparticles via Casting Method. Res. Sq..

[32] Köroğlu A., Şahin O., Kürkçüoğlu I., Ömür Dede D., Özdemir T., Hazer B. (2016). Silver Nanoparticle Incorporation Effect on Mechanical and Thermal Properties of Denture Base Acrylic Resins. J. Appl. Oral Sci..

[33] Jeon H., Kangtaek L. (2019). Effect of Gold Nanoparticle Morphology on Thermal Properties of Polyimide Nanocomposite Films. Colloids Surf. A Physicochem. Eng. Asp..

[34] Li J., Liu H., Hu Z., Wang Z., Wang B., Liu L., Huang Y., Guo Z. (2017). Flexible, Conductive, Porous, Fibrillar Polymer–Gold Nanocomposites with Enhanced Electromagnetic Interference Shielding and Mechanical Properties. J. Mater. Chem. C.

